# Assessment of the monthly risk of dirofilariosis infection in Europe and its projection to 2100 under climate change from a One Health perspective

**DOI:** 10.1186/s13071-025-07148-5

**Published:** 2025-11-27

**Authors:** Iván Rodríguez-Escolar, Alfonso Balmori-de la Puente, Elena Infante González-Mohino, Manuel Collado-Cuadrado, Elena Carretón, José Alberto Montoya-Alonso, Rodrigo Morchón

**Affiliations:** 1 Zoonotic Diseases and One Health Group, Faculty of Pharmacy, Centre for Environmental Studies and Rural Dynamization (CEADIR), University of Salamanca, Salamanca, Spain; 2 Internal Medicine, Faculty of Veterinary Medicine, Research Institute of Biomedical and Health Sciences (IUIBS), University of Las Palmas de Gran Canaria, Las Palmas de Gran Canaria, Spain; 3 Biomedical Research Institute of Salamanca (IBSAL), University of Salamanca, Salamanca, Spain

**Keywords:** Europe, Dirofilariosis, *Dirofilaria immitis*, *Dirofilaria repens*, *Culex pipiens*, *Aedes albopictus*, Ecological niche model, Number of generations of *Dirofilaria* spp., Annual infection risk, Monthly infection risk

## Abstract

**Background:**

Dirofilariosis is a vector-borne zoonotic disease primarily caused by the parasitic nematodes *Dirofilaria immitis* and *D. repens*. In Europe, the disease has expanded from traditionally endemic southern countries to central and northeastern regions, many of which are now also considered endemic. This study aimed to generate infection risk maps for dirofilariosis in Europe using ecoinformatic tools, at both annual and monthly scales, to serve as a prevention tool and contribute to more effective control of the disease, as well as helping to stop its spread.

**Methods:**

A habitat suitability map was generated for the two most important and widely distributed culicid vectors in Europe (*Culex pipiens* and *Aedes albopictus*). This map was weighted with the number of *D. immitis* generations in the vectors, both annually and monthly. The resulting annual risk map was validated with georeferenced records of *D. immitis*- and *D. repens*-infected dogs and cats. In addition, a future habitat suitability projection for both vector species was performed for the year 2100 under the Representative Concentration Pathway (RCP) 8.5 climate change scenario.

**Results:**

Dirofilariosis infection risk in Europe is highest in southern countries, where favorable climatic conditions and increased vector activity coincide. Central Europe showed medium- to high-risk values, while northern latitudes exhibited low or very low risk, correlating with lower average temperatures. Of the geolocated infected animals, 35.9%, 51% and 13% were located in high-, medium-, or low-risk areas, respectively. Infection risk appears to be very limited during winter, restricted mainly to Mediterranean coastal areas, the Canary Islands (Spain), and Madeira (Portugal); while in spring/summer it becomes high in these places and moderate across other parts of the range such us central and northeastern Europe. The 2100 projection predicts a 161.6% increase in habitat suitability for the vectors, particularly in northeastern regions, high-altitude areas, and northernmost countries.

**Conclusions:**

The combined use of habitat suitability for *Culex pipiens* and *Aedes albopictus* and the number of *Dirofilaria* spp. generations allowed the development of a more comprehensive color-coded dirofilariosis infection risk map than previously available. Monthly infection risk maps across Europe could help guide targeted prevention and control measures, disrupt disease establishment in specific areas and seasons, and raise awareness about infection risks in both animals and humans.

**Graphical Abstract:**

**Figure:**
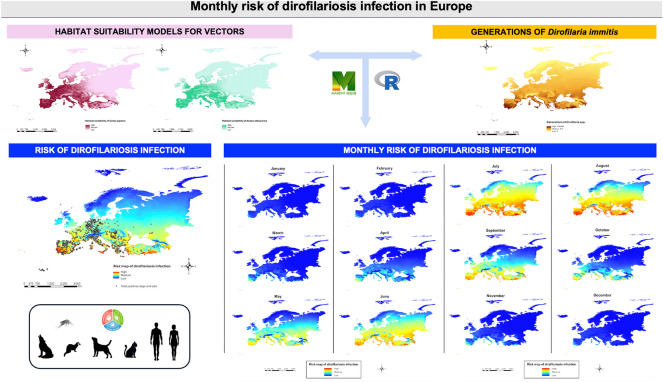

**Supplementary Information:**

The online version contains supplementary material available at 10.1186/s13071-025-07148-5.

## Background

Species of the genus *Dirofilaria* are parasitic nematodes responsible for dirofilariosis, a vector-borne zoonotic disease. The two most relevant species are *Dirofilaria immitis* (heartworm disease) and *D. repens* (subcutaneous dirofilariosis). The definitive hosts of these parasites include both domestic and wild canids and felids, with domestic dogs serving as the main reservoir. Other hosts such as domestic cats, ferrets, and wild carnivores may also be affected [1, 2], while humans are considered accidental hosts [3, 4]. Culicid mosquitoes, including species of the genera *Culex*, *Aedes*, *Anopheles*, and *Coquillettidia*, act as vectors of the parasites [5, 6].

Both *D. immitis* and *D. repens* adults release microfilariae into the bloodstream of their definitive hosts. When mosquitoes take a blood meal, they ingest these microfilariae, which then undergo two molts within the vector to develop into the infective third-stage larvae (L_3_). Upon subsequent blood feeding, the mosquito inoculates the L_3_ larvae into a new host. This developmental process is temperature-dependent, taking approximately 16–20 days at 22 °C, and only 8–10 days at 28–30 °C. Temperatures below 14 °C inhibit larval development but preserve their viability [7].

In Europe, dirofilariosis is a dynamic disease, regarded for years as emerging or re-emerging, and is currently present across much of the continent. Heartworm disease was historically endemic only in Mediterranean countries (Spain, France, Greece, Italy, Portugal, and Turkey). However, *D. immitis* has now been reported in Albania, Germany, Austria, Bulgaria, Czech Republic, Croatia, Slovakia, Hungary, Moldova, Romania, Serbia, southern Russia, and Ukraine, many of which are now considered endemic areas [8–10]. Similarly, *D. repens*, previously confined to southern and southeastern regions (e.g., Bulgaria, Spain, Greece, Italy, Romania, and Turkey), has spread over the past decade to central and northern European countries such as Germany, Austria, Belarus, Slovakia, Estonia, Hungary, Latvia, Lithuania, Poland, and Ukraine [1, 5, 9–14].

The distribution and expansion of dirofilariosis is influenced by numerous environmental and social factors, including climate change, the introduction of new vector species, increased anthropogenic activity (e.g., irrigation zones, urban development, and the creation of stagnant water bodies), enhanced transportation networks, and the greater movement of people and animals. This includes a growing number of pets traveling to endemic or imported case-reporting areas [1, 8]. Some health-based control measures include preventive treatment in domestic animals, avoidance of mosquito bites, and the diagnosis and treatment of all hosts involved [4, 15]. In addition, predictive mapping tools based on ecological niche modeling (ENM) have been developed. These models estimate infection risk by correlating vector and host presence records with environmental variables [16, 17]. ENMs have been used to model the distribution of infected hosts [18–22] and vectors involved in transmission [23, 24, 89, 90, 91, 92, 93, 94, 95], as well as to assess infection risk by integrating the potential distribution of one or more vectors and the parasite’s development within them [25–27].

Several local and national studies in Europe have estimated the risk of dirofilariosis transmission solely on the basis of temperature records [28–37]. More recently, models have integrated habitat suitability and the estimated number of annual *Dirofilaria* spp. generations in vectors in countries such as Spain, Greece, Italy, Portugal, and Serbia [25, 38–41]. At the continental level, infection risk has so far only been estimated by calculating the number of annual generations of *Dirofilaria* spp. within vectors [42, 43].

Therefore, the aim of this study was to assess the risk of dirofilariosis infection across Europe by generating colorimetric maps at both monthly and annual scales. These maps are based on ecological niche modeling of the main vectors and the estimated number of parasite generations within them. In addition, the study projects vector habitat suitability by the year 2100 under climate change scenarios. This approach seeks to provide a useful prevention and control tool for medical and veterinary professionals, pet owners, and the general public, within a One Health framework.

## Methods

### Study area: Europe

Europe covers about 10 million km^2^ in the Northern Hemisphere, bordered by the Arctic Ocean to the north, the Atlantic Ocean to the west, and the Mediterranean Sea to the south [44]. The most widespread climate according to the Köppen–Geiger classification is humid continental (Df), located in the center and east of the continent, with cold winters, cool summers, and rainfall throughout the year. Countries near the Mediterranean Sea have a Mediterranean climate (typical Mediterranean climate [Csa] and typical Mediterranean climate with warm summers [Csb]), with mild winters, dry and warm summers, and rainfall concentrated mainly in spring and autumn. Western Europe has an oceanic climate (Cfb) with cold or mild winters, cool summers, and rainfall throughout the year. In northern Europe, the predominant climate is subarctic continental (Dfc), characterized by very cold, long winters with snowfall and cold summers. Finally, in the far north, there are areas with a tundra climate (ET), where the average temperature does not exceed 10 °C in any month. Noteworthy are the Canary Islands (Spain), as well as the Azores and Madeira (Portugal), which have subtropical characteristics, with mild temperatures throughout the year, dry summers, and significant variations in rainfall [45, 46].

### Presence data

Geolocated presence points for the dirofilariosis vectors *Cx. pipiens* (8691) and *Ae. albopictus* (19,878) were obtained from: (1) the Global Biodiversity Information Facility (GBIF) data repository [47]; (2) the European Network for Medical and Veterinary Entomology of the European Centre for Disease Prevention and Control [48]; and (3) additional reported data [38, 49–56]. These vectors are the most important and widely distributed culicid mosquito species on the European continent [8, 57]. These data were processed to avoid spatial autocorrelation biases in the abundance and distribution of observations. A 1 km^2^ grid was superimposed, leaving only one observation per square. At the end of the process, the number of presence points was 2502 for *Cx. pipiens* and 2378 for *Ae. albopictus* (Supplementary Fig. S1).

### Bioclimatic and environmental variables

In total, 19 bioclimatic variables (1970–2000) related to temperature and precipitation were downloaded from the WorldClim climate database [58], both for the present and for projections to 2100 under a climate change scenario. To avoid cross-correlation and improve model calibration, a multicollinearity test was performed in R software [59] using Pearson’s correlation coefficient. Variables with a correlation coefficient equal to or greater than 0.8 were discarded [60]. After this analysis, the selected variables were: annual mean temperature (BIO_1_), isothermality (BIO_3_), temperature seasonality (BIO_4_), mean temperature of wettest quarter (BIO_8_), mean temperature of driest quarter (BIO_9_), annual precipitation (BIO_12_), and precipitation seasonality (BIO_15_). In addition, a series of environmental variables important for vector survival were also downloaded: human footprint (built environment, population density, electrical energy infrastructure, cropland, grazing land, roads, railways, and waterways) [61], rivers, water bodies, irrigated crops [62], and shrub and herbaceous density [63]. All downloaded variables had a resolution of 1 km^2^ per pixel and were processed in ArcMap 10.8 software to crop them to the same extent (the European continent) and give them the same coordinates (GCS_WGS_1984).

### Ecological niche models

To generate habitat suitability models for *Cx. pipiens* and *Ae. albopictus*, the MaxEnt algorithm [64] was used, automated by the Kuenm package [60] in R software [59]. MaxEnt calculates the habitat suitability of species on the basis of their climatic and environmental requirements, and Kuenm generates all possible models with parameter combinations and selects the best one, taking into account statistical significance (partial receiver operating characteristic [ROC] < 0.05) with 100 iterations and 50% of the presence data used for bootstrapping, the omission rate (OR = 5%), and complexity using the Akaike information criterion. A total of 119 models were generated for each species by combining the following parameters: a single set of variables, 17 regularization multiplier values “M” (0.1–1.0 at intervals of 0.1; 2–6 at intervals of 1, 8, and 10), and seven possible combinations of three feature classes “F” (linear, quadratic, and product). All generated models were validated with the mean ratio of the area under the curve (AUC), using independent occurrence points (80% for training and 20% for testing). The final habitat suitability models for *Cx. pipiens* and *Ae. albopictus*, selected on the basis of the best performance according to Kuenm’s criteria, were generated again using the clamping extrapolation option, obtaining ten replicates with the same combination of parameters chosen in the previous step. The pixels composing the habitat suitability maps for both species of culicid mosquitoes were weighted in the same proportion (50–50) using the following formula, where ENMc and ENMa are the habitat suitability values for *Cx. pipiens* and *Ae. albopictus* models, respectively:

$${\text{ENM weighted}} = \frac{{{\text{ENMc*}}50 + {\text{ENMa*}}50}}{100}$$

### *Dirofilaria* spp. generations

We calculated the number of generations of *Dirofilaria* spp. per year and in each of the 12 months of the year using the method described by Rodríguez-Escolar et al. [25] and Genchi et al. [42] based on our own R script. This methodology adds up the degrees Celsius (growing degree days or GDDs) in which the average daily temperature exceeds 14 °C, the minimum threshold necessary for the development of L_3_ larvae of the parasite in the mosquito (extrinsic incubation). A complete generation requires at least 130 GDDs within the mosquito’s lifespan, within a maximum of 30 days. The number of generations of *Dirofilaria* spp. per year were calculated by averaging the generations contained in each of the 12 months. To perform these calculations, the most up-to-date variables of average daily temperature across the continent from 1990 to 2016 were downloaded [65].

### *Dirofilaria* spp. risk map and its validation

The habitat suitability model for both species was weighted by the number of generations of *Dirofilaria* spp. in a year and in each of the 12 months in the same proportion (50–50), obtaining an average infection risk map for the whole of Europe and, in addition, a risk map for each month of the year, using the following formula:

$$Risk map = \frac{ENM weighted*50 + Generations of Dirofilaria*50}{{100}}$$

To validate the average infection risk map, we used the Natural Jenks classification method (breaks) in ArcMap with five risk classes (“very high,” “high,” “medium,” “low,” and “very low”). The georeferenced points of dogs and cats infected with *D. immitis* or *D. repens* (5408) were obtained from myVBDmap [66] and other scientific reports [8, 41, 57, 67–74] and superimposed on the average risk map.

### Projection to the year 2100 and rank-change analysis

To make the projection for the year 2100, the ENMs for both vectors were generated again using the same parameters selected for the models generated today but incorporating the projections of the bioclimatic variables for the period 2081–2100. The HadGEM3-GC31-LL model was used to analyze the effect of climate change under the Representative Concentration Pathway (RCP) 8.5 scenario [75, 76], one of the most widely used and evaluated general circulation models (GCM) in ecological niche modeling studies, as it adequately captures important climate patterns for Europe [77, 78]. The current model (present) and the model for the year 2100 (future) were transformed into binary maps of presence and absence using the 10th percentile threshold of the current map. Finally, a range change analysis was performed using the R package biomod2, which calculates the percentage of pixels that gain or lose habitat suitability for both vectors in the year 2100 compared with the current model [79].

## Results

### Habitat suitability models for *Cx. pipiens* and *Ae. albopictus*

**Fig. 1:**
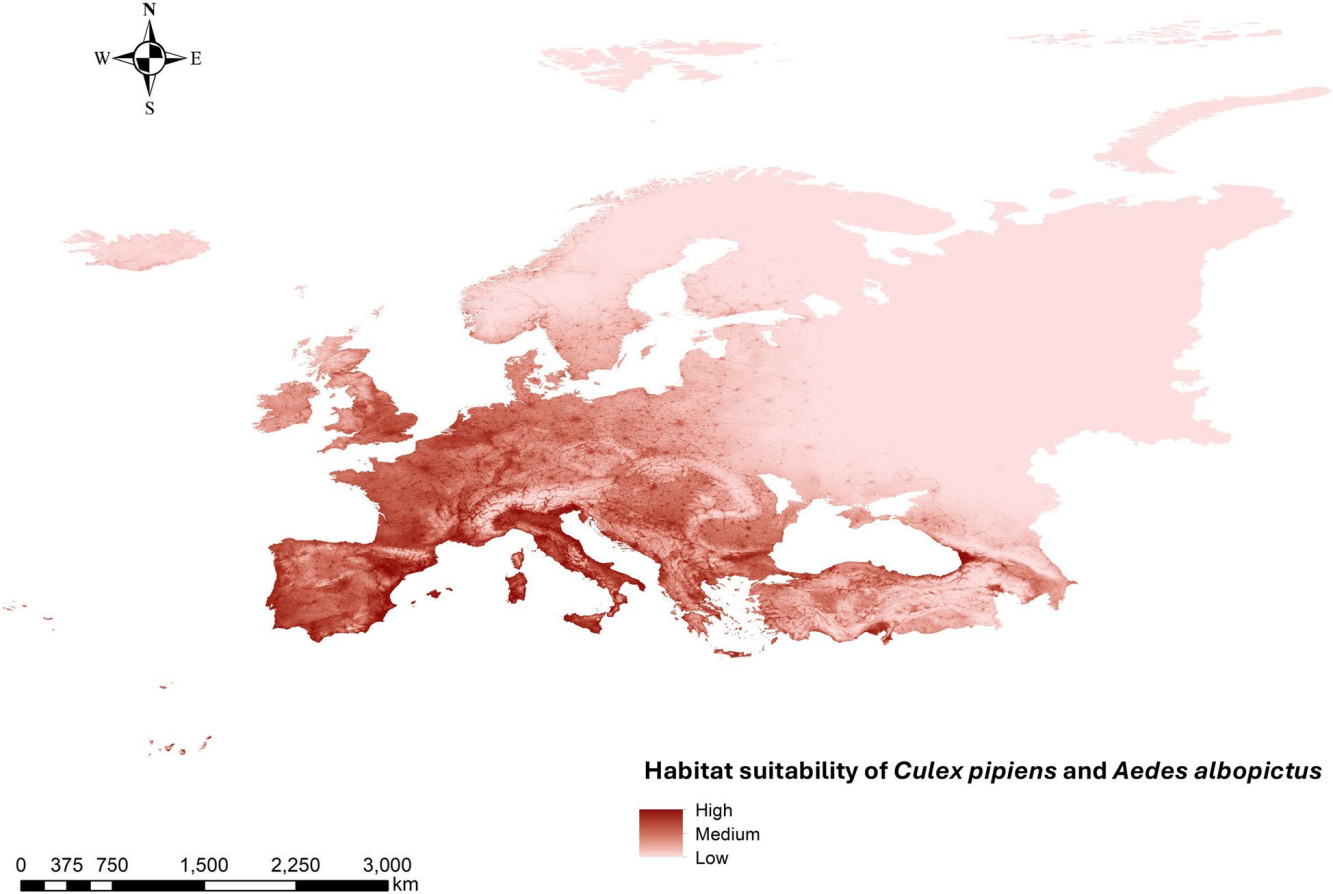
Weighted (50–50) habitat suitability map (ecological niche model) for *Culex pipiens* and *Aedes albopictus* in Europe

Out of the 119 habitat suitability models generated for each vector, the model M_0.3_F_lqp (AUC = 0.828) was selected for *Cx. pipiens*, and M_0.1_F_lp (AUC = 0.877) for *Ae. albopictus*, as they best met the Kuenm selection criteria (Supplementary Fig. S2). Regarding variable contribution, for the *Cx. pipiens* habitat suitability model, the most influential variables were BIO_4_ (temperature seasonality), water bodies, and human footprint, contributing 22.7%, 22.7%, and 20.9%, respectively. In the case of the *Ae. albopictus* model, the top contributing variables were human footprint (20%), BIO_1_ (mean annual temperature, 16.6%), and water bodies (13.3%). The least influential variables in both models were river proximity and shrubland density (Table 1). Using an equal weighting scheme (50–50), the two models were combined to generate a final habitat suitability map for both vectors. The most suitable habitats were located in areas with higher average temperatures, coastal regions, river-adjacent zones, urban environments, and irrigated agricultural lands. High suitability was observed across southern countries (Spain, Portugal, France, Italy, and Greece), as well as along the Adriatic and Black Sea coasts, southwestern UK, the Belgian and Dutch coastlines, and areas near major rivers in central Europe (Fig. 1).

**Table 1:** Percentage contribution of the variables selected in the ecological niche model for *Cx. pipiens* and *Ae. albopictus*

	Percentage contribution	
Variable	*Culex pipiens (%)*	*Aedes albopictus (%)*
Water bodies	22.7	13.3
Temperature seasonality (BIO_4_)	22.7	13.1
Human footprint	20.9	20
Annual mean temperature (BIO_1_)	16	16.6
Isothermality (BIO_3_)	6.5	11.4
Mean temperature of driest quarter (BIO_9_)	4.2	9.8
Precipitation seasonality (BIO_15_)	2.7	4
Annual precipitation (BIO_12_)	1.7	2.7
Mean temperature of wettest quarter (BIO_8_)	0.7	6.3
Herbaceous density	0.7	1
Shrub density	0.5	0.23
Irrigated crops	0.5	1.4
Rivers	0.1	0.2

### Number of extrinsic generations of *Dirofilaria* spp.

Supplementary Fig. S3 shows the average annual number of *Dirofilaria* spp. extrinsic generations in vectors across Europe. The highest number of generations (> 4) occurred mainly in southern European regions, such as the southwestern Iberian Peninsula and the archipelagos of the Canary Islands, Azores, and Madeira (Spain and Portugal), as well as along Mediterranean coastal areas—including the Levantine coast and Balearic Islands (Spain), Sicily and Sardinia (Italy), the Aegean coastline and islands (Greece), and the southeastern coast of Anatolia (Turkey). The lowest number of generations was found in mountainous areas and in northern Europe.

**Fig. 2:**
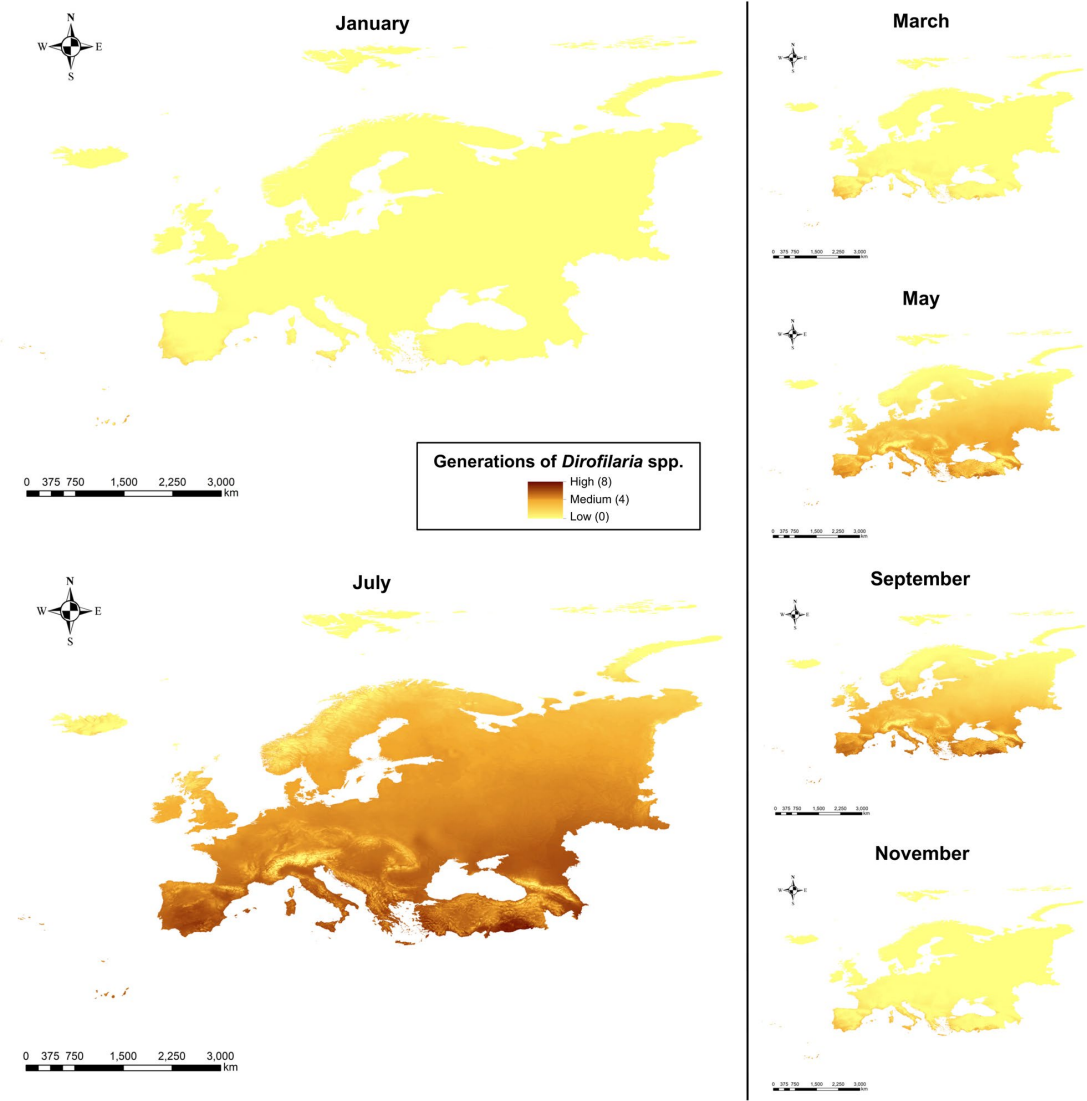
Number of generations of *Dirofilaria* spp. in Europe every 2 months (January, March, May, July, September, and November)

Regarding the monthly generation maps (Fig. 2), July showed the greatest number of locations with a high number of generations, with very high values (> 8) across Mediterranean countries (Spain, Portugal, southern France, Italy, Greece, and Turkey), and moderate-to-high values across most of the continent, except in northern latitudes and high-altitude mountain areas. In contrast, January had the lowest generation numbers, reaching zero across nearly all of Europe, except for some coastal Mediterranean zones.

### Annual infection risk map for dirofilariosis and its validation

**Fig. 3:**
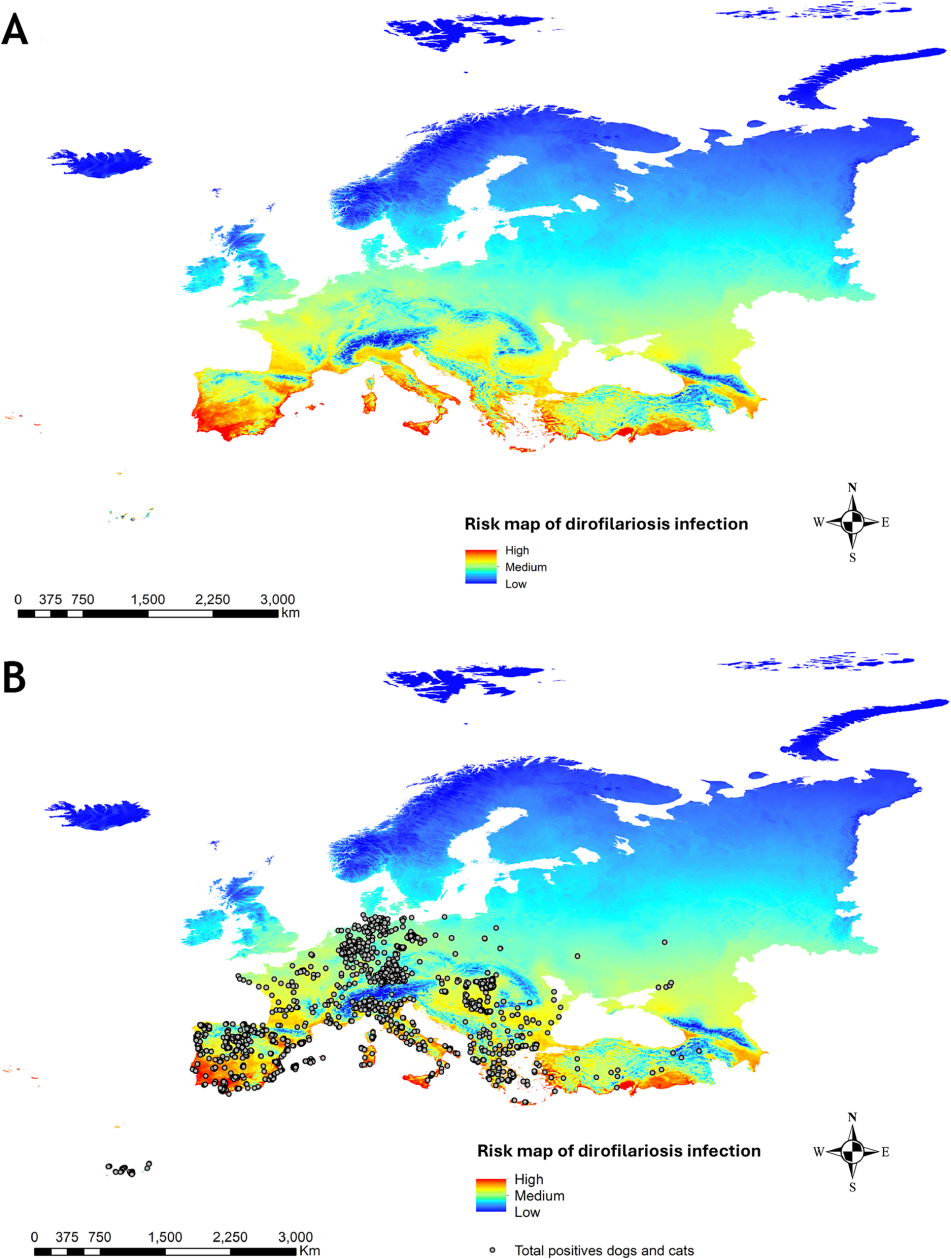
**A** Risk map of dirofilariosis infection in Europe and **B** with the georeferenced points of dogs and cats infected with *D. immitis* or *D. repens*

The resulting map from the weighted combination of both vector ENMs and the average generation map for *Dirofilaria* spp. is shown in Fig. 3a. This map represents the average annual risk of dirofilariosis infection across Europe. The highest infection risk areas were mainly located in southern countries, such as Spain and Portugal—particularly in the southwestern Iberian Peninsula, the Levantine coast, the Ebro Basin, the Balearic and Canary Islands (Spain), and the Azores (Portugal). Other high-risk regions included the French Riviera, the Italian coastline (including Sicily and Islands of Sardinia) with the Po Valley, Albania, the Aegean region, and southwestern Turkey. Central Europe presented medium-to-high infection risk values, whereas northern latitudes (e.g., the Scandinavian Peninsula, northern UK), characterized by lower temperatures, displayed low or very low infection risk. The validation of the model (Fig. 3b), using 5408 georeferenced infection cases, showed that 35.9% occurred in high/very high-risk zones, 51% in medium-risk zones, and 13% in low-risk zones.

### Monthly infection risk maps for dirofilariosis

**Fig. 4:**
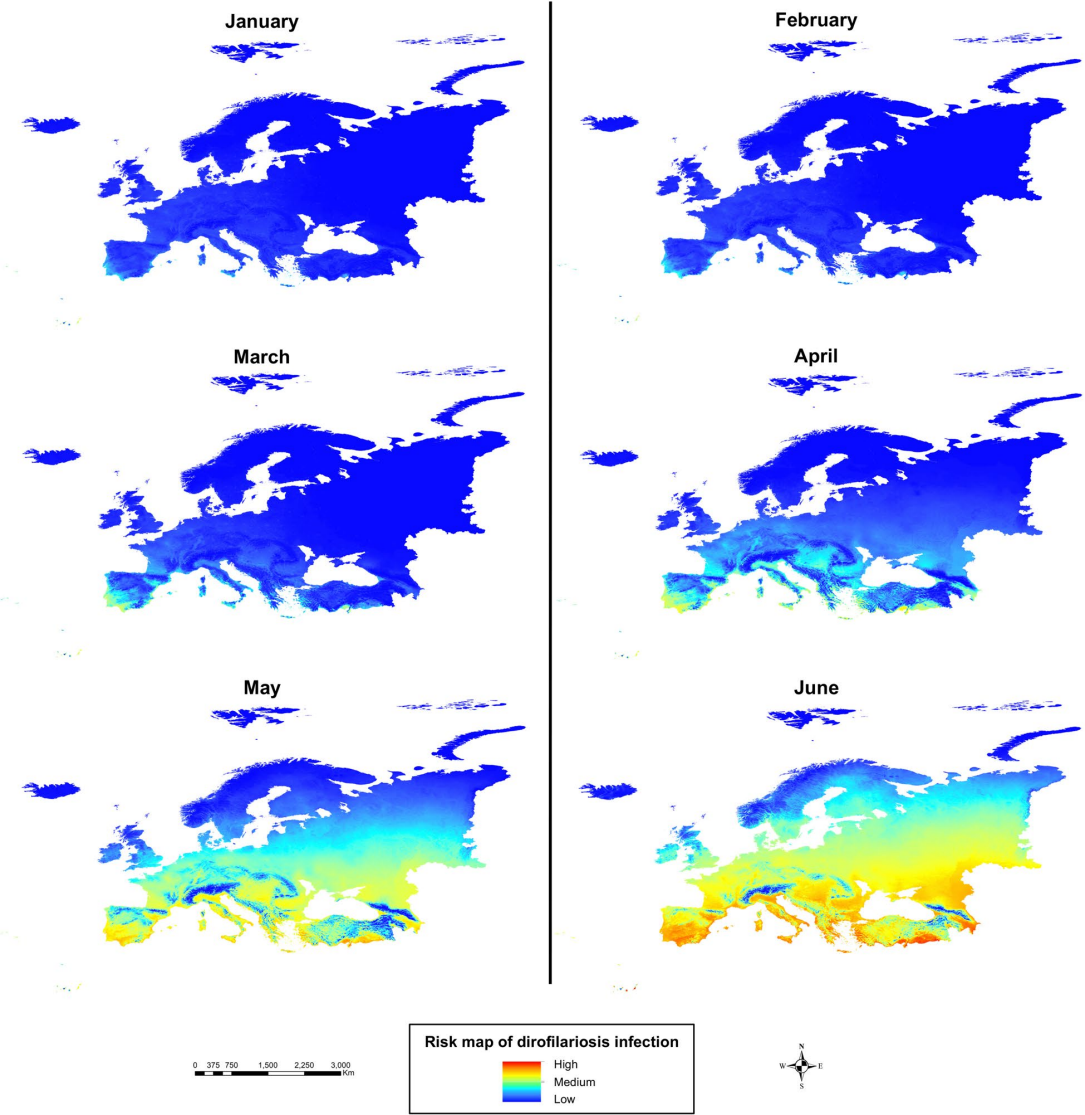
Risk map of dirofilariosis infection in Europe from January to June (first 6 months of the year)

**Fig. 5:**
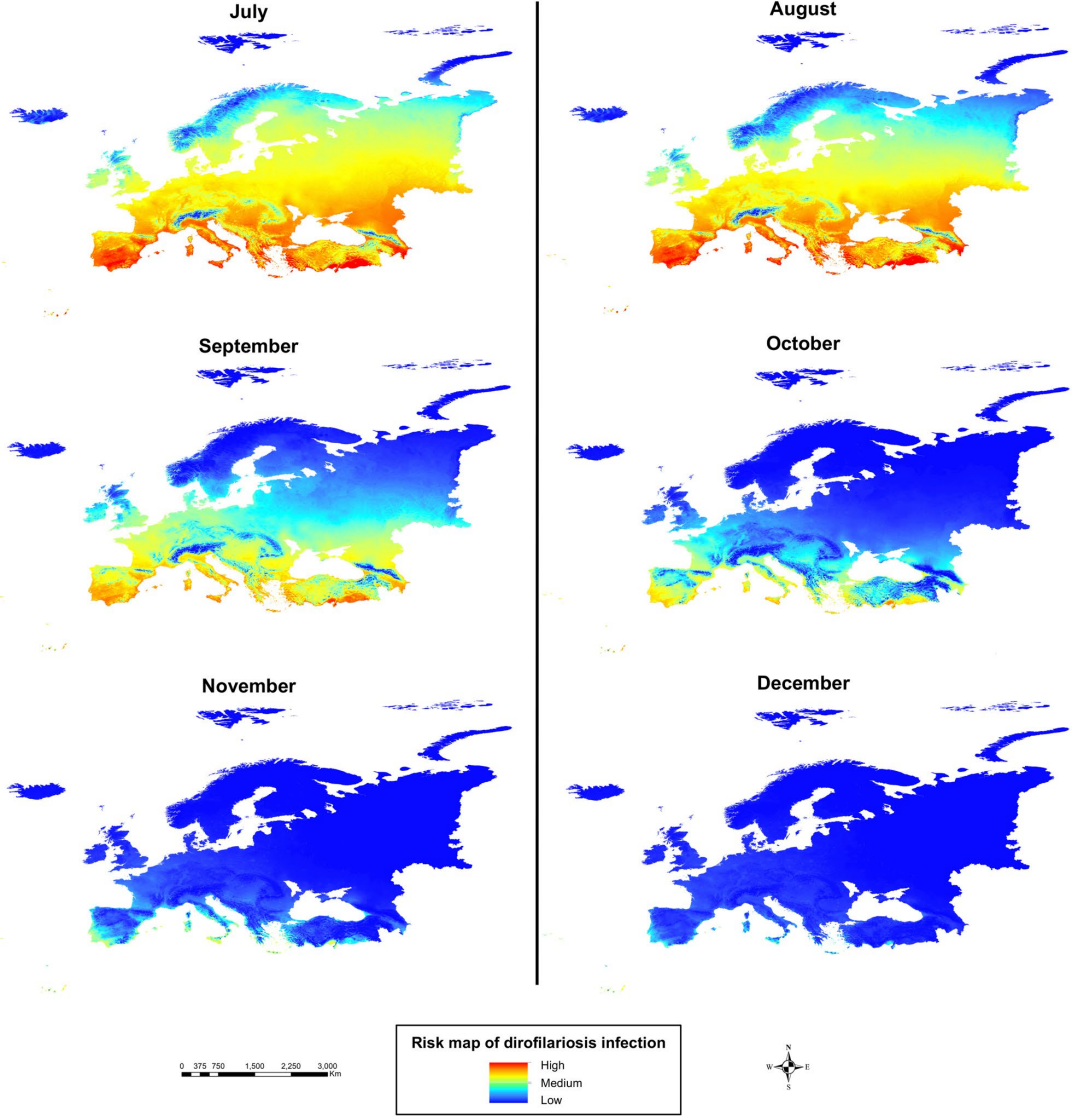
Risk map of dirofilariosis infection in Europe from July to December (last 6 months of the year)

Analysis of monthly risk maps throughout the year (Fig. 4) revealed that during the winter months, infection risk was very low across almost the entire continent, except for some coastal areas of Mediterranean countries (Spain, Portugal, Italy, Greece, and France). In spring, infection risk gradually increased from south to north. In summer (Fig. 5), infection risk rose significantly across most of the continent, except at higher latitudes (northern UK, northern Russia, and Scandinavia) and in mountainous areas. Southern Europe, with its warmer climate, showed very high infection risk values, while central Europe ranged from moderate to high risk. Finally, during autumn, infection risk declined gradually from north to south.

### Projection of habitat suitability for vectors in 2100

**Fig. 6:**
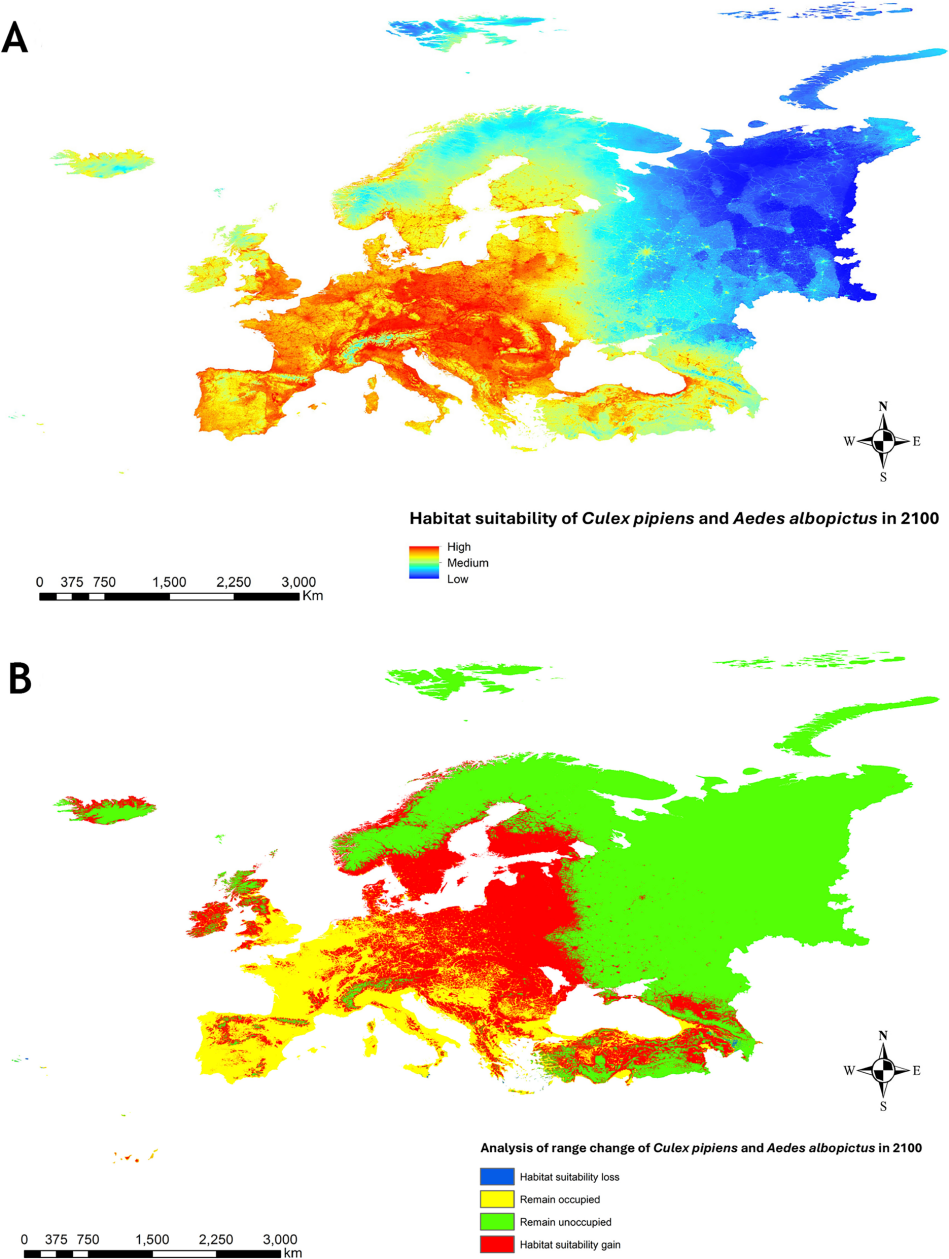
**A** Projection of weighted (50–50) habitat suitability for *Cx. pipiens* and *Ae. albopictus* in Europe for the year 2100 under the RCP 8.5 climate change scenario. **B** Range-change analysis of weighted (50–50) range change for *Cx. pipiens* and *Ae. albopictus* in Europe for the year 2100, showing areas of gain and loss and those that remain unchanged in terms of habitat suitability

The future projection of habitat suitability for *Cx. pipiens* on 2100, under a climate change scenario, generated a gain of 84.89% and a loss of 0.11%. For *Ae. albopictus*, the gain in habitat suitability was 336.13% and the loss was 1.06% (Supplementary Fig. S4). The future projection for both species (Fig. 6a) shows a considerable increase in habitat suitability for both vectors. The range-change analysis for each species is shown in Supplementary Fig. S5 and for both species in Fig. 6b. The latter indicates a 161.6% increase in suitability at the European territory level. This increase occurs mainly in the north-eastern regions of the continent, at higher latitudes, and in higher altitude areas, which currently have cold climates and low suitability for the presence of these vectors. In the rest of the territories, the risk remains stable.

## Discussion

In Europe, dirofilariosis has continued to expand over recent decades [3–5, 8]. Several factors are associated with this expansion, including climate change, the increasing number of pet travel, and the lack of preventive actions in animal hosts [8, 15].

Studies aiming to assess the infection risk of dirofilariosis in Europe as a control strategy are still scarce. Some have relied exclusively on temperature records, often assuming an oceanic climate throughout Western Europe with sufficient humidity and warmth for the parasite to develop within the vector [30, 35, 36, 38, 42, 43, 80]. Other studies, conducted at national or regional scales in Spain, Portugal, Italy, Serbia, and Greece, have integrated habitat suitability models for one vector species with the estimated development of *Dirofilaria* spp. within that vector [25, 39–41]. However, these efforts remain geographically limited and fail to provide an integrated overview of the infection risk across the entire European continent.

In our study, we evaluated the potential risk of *Dirofilaria* spp. infection across Europe by generating a color-coded map at both annual and monthly scales. This was carried out by modeling the habitat suitability of the two most relevant and widely distributed vector species in Europe (*Culex pipiens* and *Aedes albopictus*) using ecological niche models (ENMs), and weighting these maps by the estimated number of parasite generations within the vector. This approach represents a significant advancement, as it yields temporally targeted depiction of the current infection risk across Europe. In addition, the high spatial resolution (~1 km^2^) allows for the development of more localized prevention strategies.

The ENMs developed for both vector species yielded high AUC values (> 0.8), indicating strong predictive performance in discriminating between presence locations and background environmental conditions. The most influential variables in the distribution of *Cx. pipiens* were temperature seasonality (BIO_4_) and proximity to water bodies (22.7% each), while for *Ae. albopictus*, human footprint and mean annual temperature (BIO_1_) were the main contributors (≥ 16.6% each). This confirms the pivotal role of temperature-related variables in vector survival, with low precipitation acting as a limiting factor for mosquito establishment [8, 81, 82]. However, the negative effects of low rainfall are often compensated by the presence of natural and artificial water bodies, irrigated agricultural zones, and urban areas that provide suitable habitats for vector reproduction—areas where previous studies have reported high prevalence of canine dirofilariosis [74]. In the case of *Ae. albopictus*, the high contribution of human footprint as a predictor variable aligns with its well-known anthropophilic and urban-adapted behavior [41, 83]. Therefore, it is not surprising that aside from temperature-related factors, water availability and human activity are among the main contributors to the habitat suitability of *Cx. pipiens* and *Ae. albopictus*, respectively.

With regard to the continental risk map for dirofilariosis, high-risk zones were mainly located in areas with elevated temperatures, both natural and artificial water sources, and high human footprint—settings where dogs and humans coexist closely, increasing the zoonotic infection potential [1, 8]. Conversely, colder regions and mountainous zones presented low risk values, in line with the limited vector presence reported in such areas. Monthly infection risk maps revealed seasonal variation, with very low risk during winter months except for some coastal Mediterranean areas. Risk increased progressively throughout spring, reaching its peak in summer across most of Europe—except in the highest latitudes and mountainous areas—consistent with the seasonal dynamics of the disease described in previous studies [28, 42, 43].

Our projection for the year 2100 suggests a 161.6% increase in habitat suitability for both vector species as a result of climate change. This projection indicates a potential spread of *Cx. pipiens* and *Ae. albopictus* into northeastern Europe, more northern latitudes, and mountainous regions—areas that were previously nonendemic—unless control measures are implemented. Climate change appears to influence the thermal limits for transmission, which may substantially alter the seasonal patterns and geographic distribution of zoonotic diseases in cooler countries, as reflected in our findings [35, 84–88].

An additional factor that may influence the distribution and persistence of dirofilariosis, particularly in southern Europe, is the growing population of stray or unowned dogs and cats. These animals often remain outside veterinary surveillance programs and do not receive prophylactic treatment, enabling the maintenance of local transmission cycles even in areas where preventive measures are widely applied to owned pets. Their presence in urban and periurban environments, frequently overlapping with high-risk zones identified on our maps, may thus contribute to sustaining parasite circulation and reintroduction after control efforts. Incorporating data on stray animal populations and their infection rates into future models would improve the accuracy of risk assessment and help design more effective One Health-based intervention strategies.

Among the limitations of our study, the vector component should be highlighted first. The present records compiled from GBIF/VectorNet and literature are subject to spatial sampling biases and temporal heterogeneity. Furthermore, the 50–50 weighting of the two vector ENMs implicitly assumes an equal contribution across space and seasons, whereas vector competition, urban versus rural bionomics, and diapause vary geographically. Second, the thermal development module uses generalized thresholds (≥ 14 °C; ≥ 130 GDD in ≤ 30 days) and does not explicitly account for humidity, vector longevity, microclimatic effects (e.g., urban heat islands), or nonlinear thermal responses. Third, environmental layers and different time bases may smooth microhabitats and introduce temporal mismatch. Fourth, validation using georeferenced cases of dogs and cats is limited by underreporting, diagnostic variability, uncertainty about the timing of infection, travel history, and geolocation inaccuracies; consequently, monthly risk could not be empirically validated. Finally, climate projections were based on a single global climate model and a high emissions pathway, which does not reflect structural and scenario uncertainty. These caveats delineate priorities for future work, including multivector ENMs with biased background correction, integration of host and intervention layers, joint climate projections, and prospective entomological and serological validation with monthly resolution.

## Conclusions

This study presents the first Europe-wide assessment of *Dirofilaria* spp. infection risk. The spatiotemporal maps show a clear seasonal pattern, with risk increasing from winter to summer and peaking in July, particularly in southern and Mediterranean regions where conditions favor vector and parasite development. These maps provide a valuable tool for guiding surveillance, diagnosis, and control of dirofilariosis in Europe. Their application supports targeted prevention, prioritization of intervention areas, and incorporation of the One Health approach into the management of this emerging vector-borne zoonosis.

## Supplementary Information

Additional file 1 (Supplementary Figure 1. Geolocated points of Cx. pipiens and Ae. albopictus in Europe.)

Additional file 2 (Supplementary Figure 2. Habitat suitability map (Ecological niche model) for Culex pipiens (A) and Aedes albopictus (B) in Europe.)

Additional file 3 (Supplementary Figure 3. Number of extrinsic generations of Dirofilaria spp. in Europe.)

Additional file 4 (Supplementary Figure 4. Projection of habitat suitability of Cx. pipiens (A) and Ae. albopictus (B) in Europe for the year 2100 under the RCP 8.5 climate change scenario.)

Additional file 5 (Supplementary Figure 5. Range change Analysis of range change of Cx. pipiens (A) and Ae. albopictus (B) in Europe for the year 2100 showing areas of gain, loss and those that remain unchanged in terms of habitat suitability.)

## Data Availability

Data supporting the main conclusions of this study are included in the manuscript.

## Abbreviations

*Ae. Albopictus*: *Aedes albopictus*
AUC: Area under the curve
BIO_1_: Annual mean temperature
BIO_3_: Isothermality
BIO_4_: Temperature seasonality
BIO_8_: Mean temperature of wettest quarter
BIO_9_: Mean temperature of driest quarter
BIO_12_: Annual precipitation
BIO_15_: Precipitation seasonality
Cfb: Oceanic climate
Csa: Typical Mediterranean climate
Csb: Typical Mediterranean climate with warm summers
*Cx. pipiens*: *Culex pipiens*
*D. immitis*: *Dirofilaria immitis*
*D. repens*: *Dirofilaria repens*
Df: Humid continental climate
Dfc: Subarctic continental climate
ENM: Ecological niche model
ENMa: Habitat suitability model for *Ae. albopictus*
ENMc: Habitat suitability model for *Cx. pipiens*
ET: Tundra climate
GDDs: Growing degree days
