# Supplementary figures and images for: Assessment of the monthly risk of dirofilariosis infection in Europe and its projection to 2100 under climate change from a One Health perspective
Source: Parasit Vectors. 2025 Nov 27;18:516. doi: 10.1186/s13071-025-07148-5 (PMC12751232; doi:10.1186/s13071-025-07148-5)

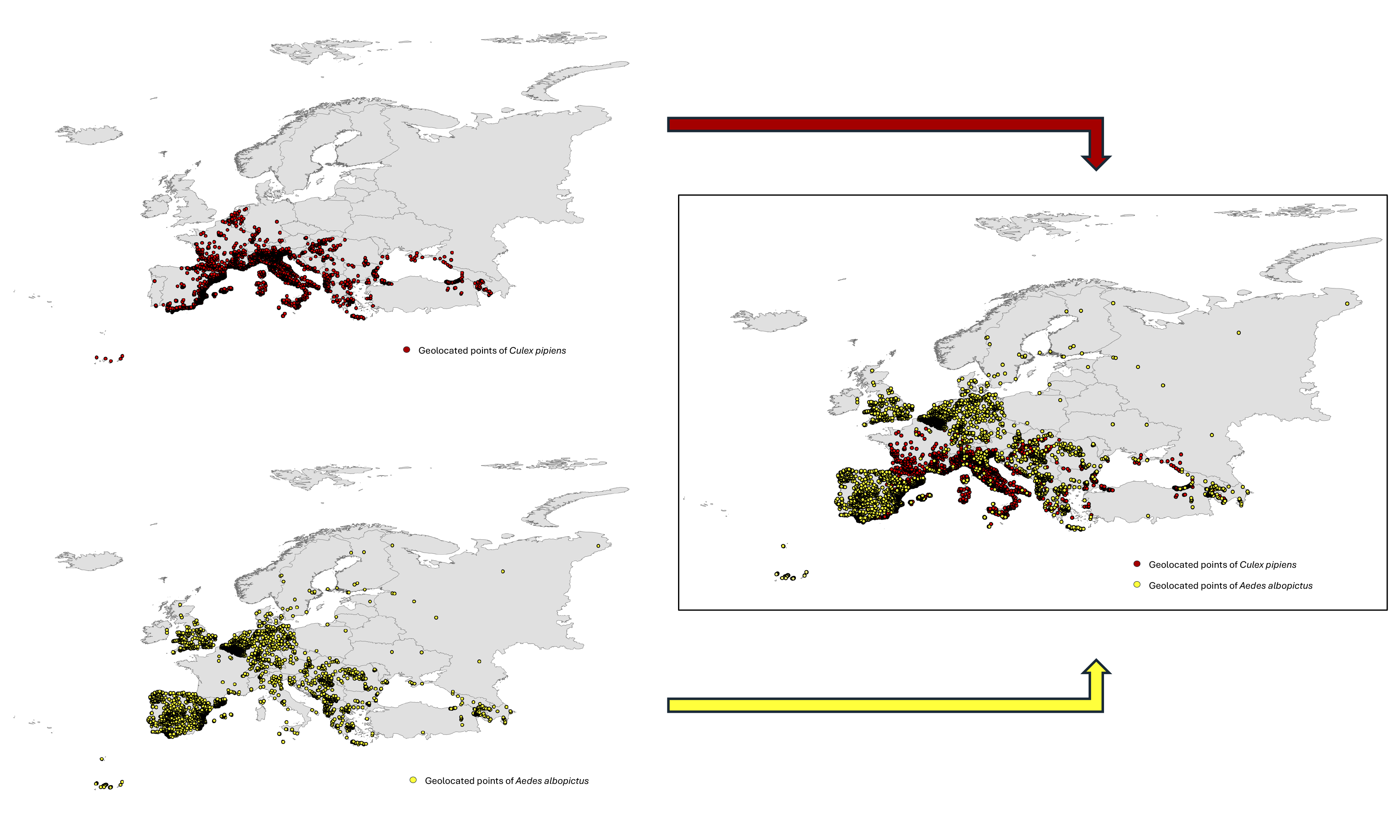

Supplement: Supplementary file 1 — Additional file 1 (Supplementary Figure 1. Geolocated points of Cx. pipiens and Ae. albopictus in Europe.) [file 13071_2025_7148_MOESM1_ESM.tif]

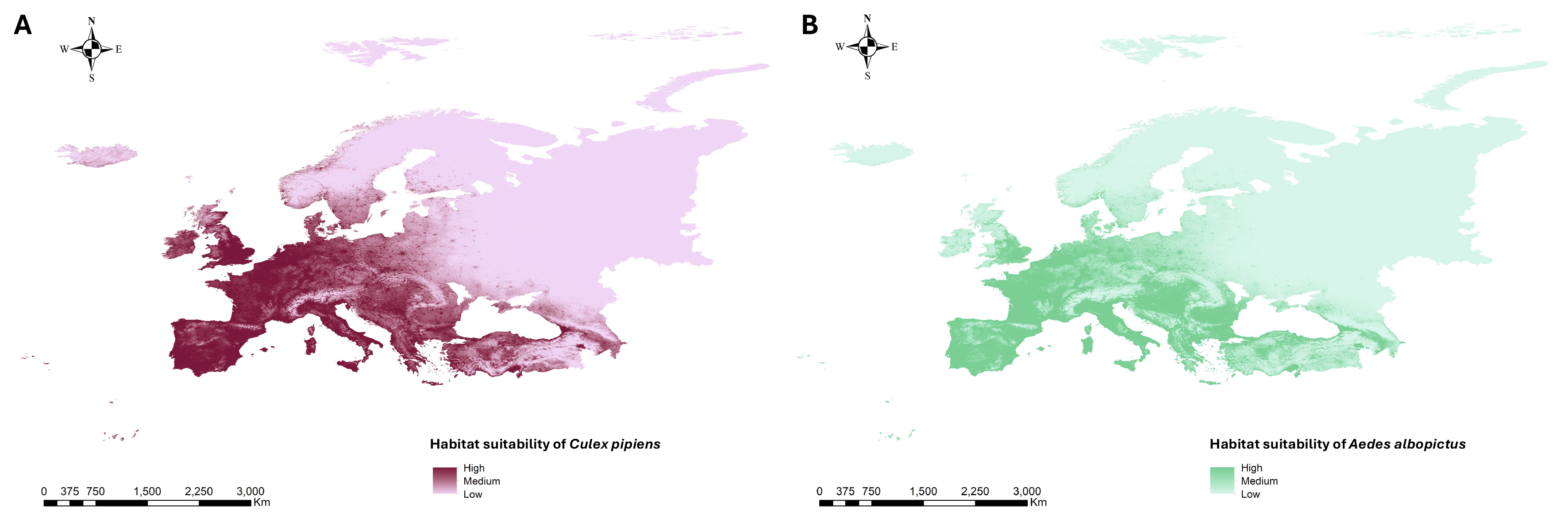

Supplement: Supplementary file 2 — Additional file 2 (Supplementary Figure 2. Habitat suitability map (Ecological niche model) for Culex pipiens (A) and Aedes albopictus (B) in Europe.) [file 13071_2025_7148_MOESM2_ESM.tif]

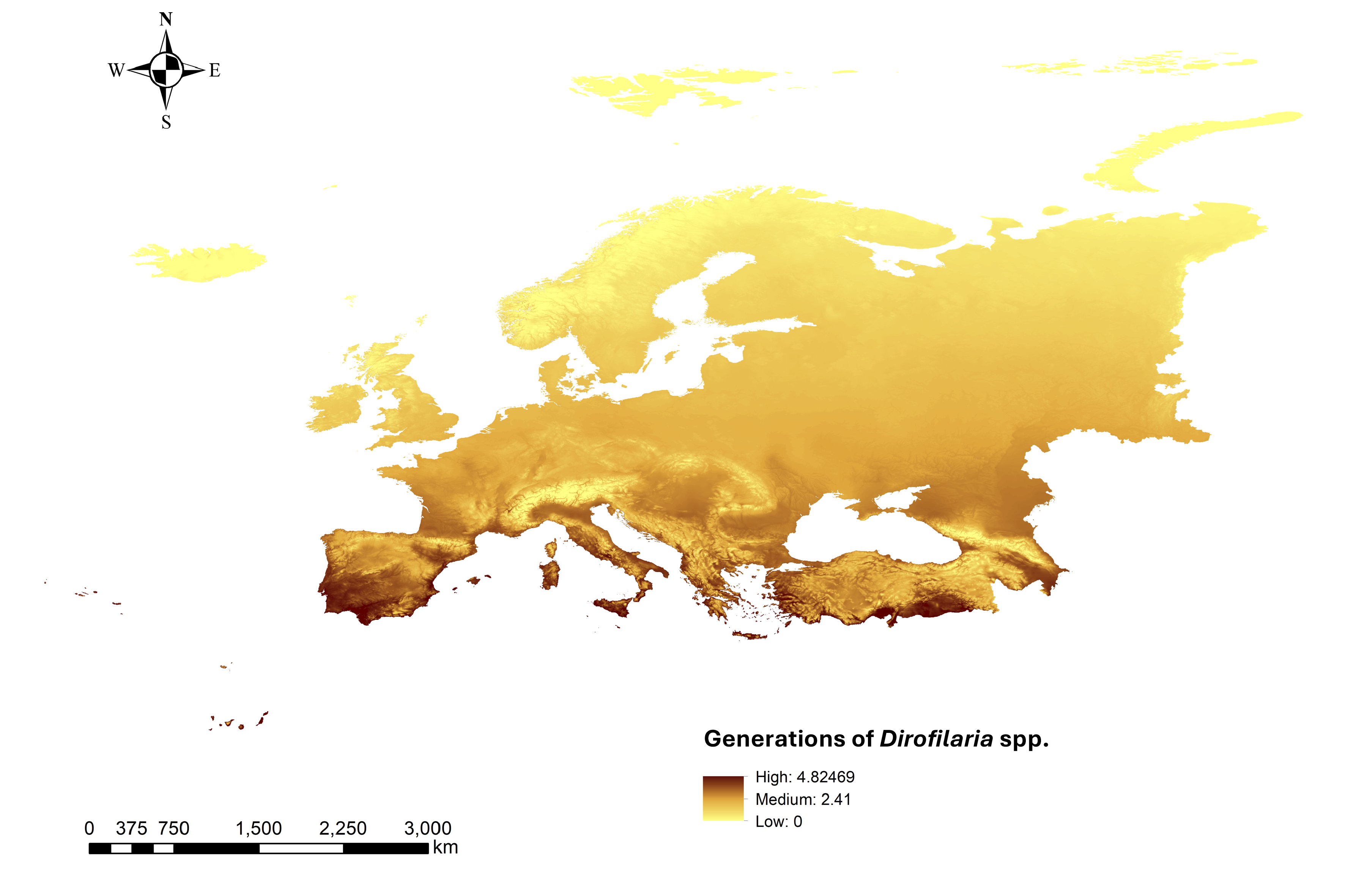

Supplement: Supplementary file 3 — Additional file 3 (Supplementary Figure 3. Number of extrinsic generations of Dirofilaria spp. in Europe.) [file 13071_2025_7148_MOESM3_ESM.tif]

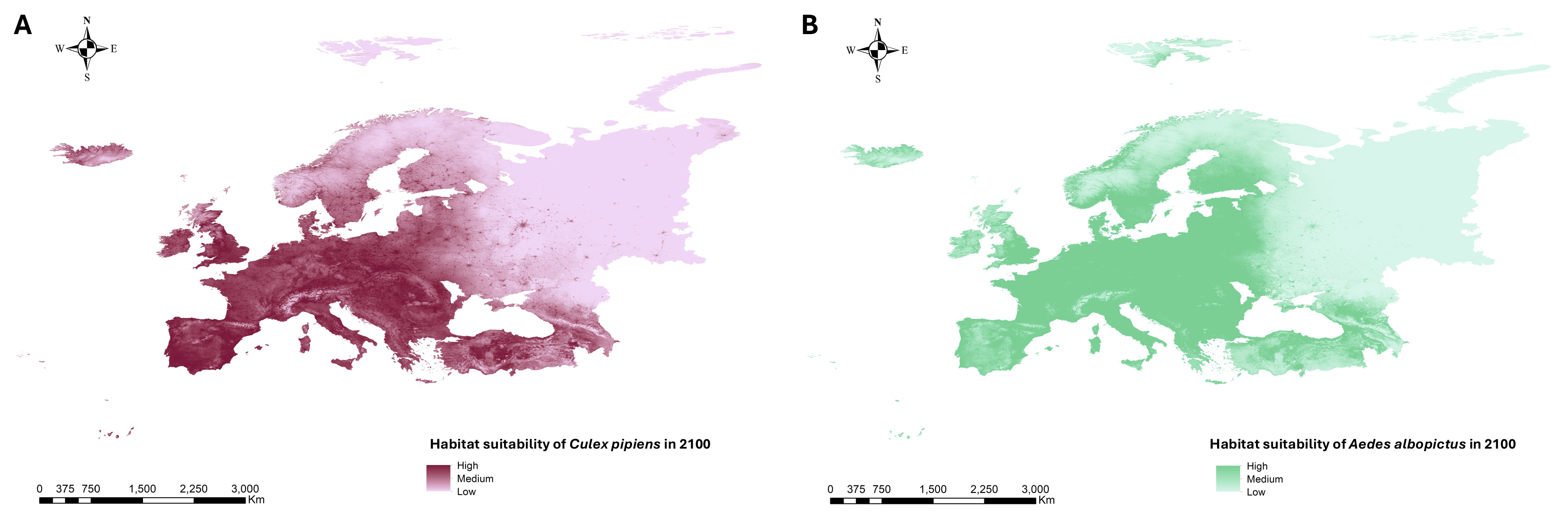

Supplement: Supplementary file 4 — Additional file 4 (Supplementary Figure 4. Projection of habitat suitability of Cx. pipiens (A) and Ae. albopictus (B) in Europe for the year 2100 under the RCP 8.5 climate change scenario.) [file 13071_2025_7148_MOESM4_ESM.tif]

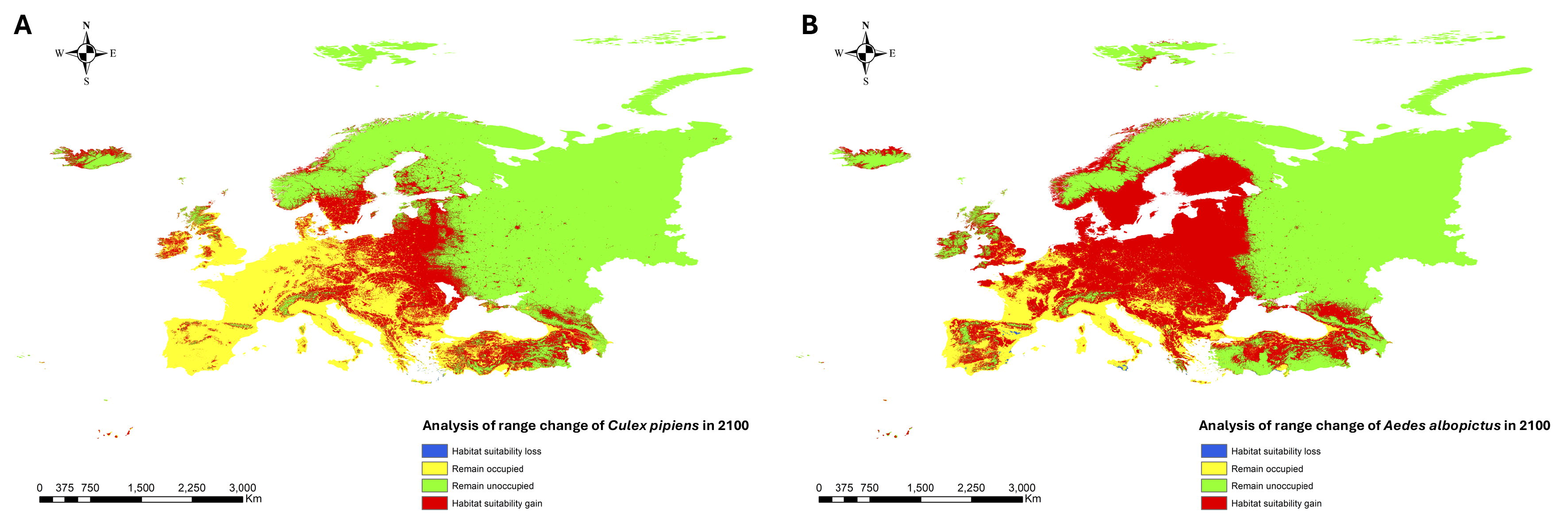

Supplement: Supplementary file 5 — Additional file 5 (Supplementary Figure 5. Range change Analysis of range change of Cx. pipiens (A) and Ae. albopictus (B) in Europe for the year 2100 showing areas of gain, loss and those that remain unchanged in terms of habitat suitability.) [file 13071_2025_7148_MOESM5_ESM.tif]
